# Influence of Glutathione and Ascorbic Acid Treatments during Vinification of Feteasca Regala Variety and Their Antioxidant Effect on Volatile Profile

**DOI:** 10.3390/bios9040140

**Published:** 2019-12-05

**Authors:** George Adrian Cojocaru, Arina Oana Antoce

**Affiliations:** University of Agronomic Sciences and Veterinary Medicine of Bucharest, Faculty of Horticulture, Laboratory of Enology, 59 Marasti Ave., Sector 1, 011464 Bucharest, Romania; cojocaru.george@ymail.com

**Keywords:** e-Nose, DFA, Feteasca regala, wine, volatile profile, glutathione, ascorbic acid, higher alcohols, fatty acid esters

## Abstract

Volatile profiles were determined for white wines of Feteasca regala variety produced from musts in which the antioxidants glutathione and ascorbic acid were added in different proportions before inception of alcoholic fermentation. Treatments with these antioxidants affect some volatile compound evolution and positively influence the wine volatile profile. After one year of storage in bottles with and without carbon dioxide protection the volatile profiles of the wines were assessed by using a Fast GC Alpha MOS Heracles e-Nose by applying a DFA multivariate statistical method and AroChemBase database for compound identification. The analyses showed that some higher alcohols, such as 2-phenylethanol and 2-methyl-1-butanol were in lower concentrations in wines treated with reduced glutathione, while the main ethyl fatty acid esters, such as ethyl butanoate, ethyl hexanoate, ethyl octanoate, and ethyl decanoate were better preserved when higher concentrations of any of the antioxidants were added in must. On the other hand, it was observed that some other volatile compounds were not affected by these applied treatments.

## 1. Introduction

Advanced reductive technologies for white wine production involve the use of inert gases during all stages of processing to counteract the oxygen solubilization from the atmosphere into wine, along with some treatments with certain antioxidants to ensure a good protection. Various authors have already revealed the positive effects of inert gases on white wine production [1,2,3,4,5,6] and also of certain antioxidant treatments and their combined effect during vinification, at bottling or their impact during storage [7,8,9,10,11,12,13,14,15]. The most common antioxidant treatments used on grapes and musts are sulfur dioxide and ascorbic acid, for which a comprehensive review was published in 2016 [16]. Other antioxidants often used for protection are the oenological tannins of various chemical compositions, origins, and effects against wine oxidation [17]. Tannins are mostly used in red wines [18], but have also a good effect, in lower dosages in white wine [19]. Besides these classic antioxidants reduced glutathione [20,21,22] can be added as a newer treatment with higher antioxidant capacity, recently accepted by the Organisation of Vine and Wine and included in OIV resolutions [23,24]. Furthermore, it is well known that the usage of inert gases in winemaking technology, applied along with any of the other possible antioxidant treatments, may improve the product characteristics, by further protecting the color and the volatile organic compounds forming the wine aroma. At the same time, the presence of antioxidants during the fermentation has an influence on the metabolic pathways of yeasts and speed of fermentation that usually gives the wines their specific fermentation aroma, which overlaps with the primary aromatic profile from the grape variety used as raw material [25]. In previous papers it was shown that the treatments with antioxidants on Feteasca regala variety induce detectable effects on the sensory profile [25] as well as on the general volatile profile (wine fingerprint) determined by an electronic nose based on CG principle [26]. However, the evolution of certain aroma compounds of white wines during bottle storage is of great interest [27,28] and this paper presents the differences among the volatile compounds found in the bottled wines obtained by various combinations of antioxidant treatments. The paper also attempts to identify specific compounds present in wines either from the grapes or as a result of fermentation, and to shed light on the types of antioxidant treatments which better preserve typical varietal volatile organic compounds.

## 2. Materials and Methods

For this study wines were produced from grapes of Feteasca regala variety harvested from the experimental field of the University of Agronomic Science and Veterinary Medicine of Bucharest, Faculty of Horticulture. The grapes were destemmed and crushed and the must was separated in a classical vertical hydraulic press, only free-run must being used for the experiment. The must was allowed to settle for 24 h at 10 °C. The main parameters of the must were 21.6% Brix; 82.7 meq/L total titratable acidity; pH 3.33 and 135 mg/L YAN. The clarified must was fermented in temperature-controlled conditions in stainless steel tanks of 50 L volume. The main antioxidant treatments were performed on the must before the onset of alcoholic fermentation and consisted of additions of reduced glutathione (Carl Roth GmbH, Karlsruhe, Germany, 98%) and/or ascorbic acid (Carl Roth GmbH, ≥99%, p.a.) in various concentrations. The used doses of reduced glutathione, GSH (coded G) were of 0, 20, or 40 mg/L, while the doses of ascorbic acid (A) were of 0 or 50 mg/L, thus leading to 5 main combinations, coded in accordance to the antioxidant treatments applied to musts as G00A00, G20A00, G20A50, G40A00, and G40A50 (Table 1).

The onset and a good progress of alcoholic fermentation was ensured with 20 g/hL of organic nitrogen (V Starter TF, Enologica Vason) added to achieve an optimum YAN [29] and 20 g/hL of *Saccharomyces cerevisiae* yeast inoculum (Premium blanc 12 V, Enologica Vason). After 3 weeks of fermentation the resulted wines were racked off and let for cold stabilization for 3 months in tanks.

The resulted wines were analyzed in accordance to the standard methods of the OIV [30]. The usual wine parameters were not much influenced by the antioxidant treatments, being for all samples in the range of 13.6–13.8% v/v for alcohol, 52.27–54.55 meq/L for total acidity, 3.48–3.54 pH, 1.17–2.83 g/L reducing sugar, 19.52–19.94 g/L non-reducing extract, 17.59–38.11 mg/L free sulfur dioxide, and 122.5–145.79 mg/L total sulfur dioxide.

At the end of the cold stabilization period, the experimental variants were bottled using a gravity filler in standard 75 cL green glass bottles. At this point, some supplementary treatments were performed for further antioxidant protection, consisting of addition of catechinic tannins in doses of 0 or 20 mg/L (sample codes getting a supplementary suffix of T00 or T20, respectively), thus, the number of the antioxidant treatment combination rising to 10. Furthermore, half of all the samples were also protected at bottling by filling the empty space with carbon dioxide. The samples filled with carbon dioxide were coded with an additional suffix, CO_2_, with this final treatment the number of total combinations reaching 20 (Table 1). The bottled wines were allowed to age in bottles for one year before analysis. The samples thus prepared were classified in groups to facilitate analysis and comparisons, the groups formed being: Group I—samples containing combinations of G and A, irrespective of the presence or not of either tannin or CO_2_ treatments, Group II—samples containing G, irrespective of the tannin or CO_2_ treatments, Group III—samples containing A, irrespective of the tannin or CO_2_ treatments.

The level of sulfur dioxide during the experiment was controlled in the musts and wines, being corrected whenever necessary, so that, for instance, when measured 3 months from bottling, the average of the free sulfur dioxide in wines was 26 mg/L and the total sulfur dioxide was 132 mg/L. More detailed results for the main wine parameters are presented in another paper [25].

The sample codification describing all the antioxidant treatments underwent is detailed in Table 1.

The volatile profiles were determined by using an electronic nose from Alpha MOS, France. This electronic nose is a Heracles e-nose analyzer working on the principles of fast gas chromatography. The apparatus uses a Tenax trap for pre-concentration of volatile organic compounds, which are then split and simultaneously introduced in two short capillary columns of different polarities, one being non-polar, DB5 (5% diphenyl, 95% dimethylpolysiloxane), and the other having a low/mid polarity, DB1701 (14% cyanopropylphenyl, 86% dimethylpolysiloxane). The separated volatile organic compounds are simultaneously detected by dual flame ionization detectors (FID), located at the end of each chromatographic column. The device uses hydrogen as carrier gas. The instrument running conditions are summarized in Table 2. More details regarding the methodology and the Alpha MOS Heracles analyzer can be found in other papers [31,32,33]. The apparatus is controlled by software AlphaSoft v12.42 which is also used to process the data by various statistical methods, after the data acquisition is completed. The software also integrates AroChemBase version 2010, a comprehensive database which allows for the chemical identification and sensory description of volatile compounds. The identification of the volatile compounds is based on a reference sample of standard n-alkane mixture (n-C_6_ to n-C_16_) analyzed in the same running conditions as the wine samples. Using this reference sample, the calibration and calculation of Kovats indices for the apparatus are performed. For both chromatographic columns the identification of volatile organic compounds is based on retention Kovats indices, this method being more reproducible and reliable than in the case of the retention time usage.

The statistical package of the AlphaSoft v12.42 includes the discriminant factor analysis (DFA), which was selected as the most relevant method to separate the groups of samples and to identify a possible pattern correlated with the applied antioxidant treatments. For the e-nose analysis 4 mL of wine from each variant, three repetitions each, were introduced in 10 mL vials and sealed with magnetic caps designed for autosampler HS 100 (Combi PAL Auto-Sampler System, CTC Analytics AG, Zwingen, Switzerland). The AlphaSoft v12.42 program records the chromatograms on both columns and calculates for each identified chromatographic peak an area (A) or a relative area (RA), which are all included into an integration report. For this type of electronic nose, each of the chromatographic peaks is considered a sensor and the statistical evaluation can take into account, upon user selection, all of them or only those sensors that are significantly different among the samples classified in groups. A sensor number is assigned to each peak and the column on which it was obtained is identified by a suffix, -1 for the column DB5 and -2 for the column DB1701. These sensors are actually correlated with certain volatile organic compounds found in the analyzed samples and some of them can be identified by using the integrated chemical compound database, AroChemBase. Identification of each volatile organic compound was carried out by comparing Kovats retention indices of samples with those in AroChemBase library.

Before performing multivariate statistical analysis, on a case-by-case basis, the most appropriate selection of sensors is decided, taking into account the “discrimination power” calculated by AlphaSoft v12.42 for each sensor. After selecting the most relevant discriminative sensors for a set of samples, an extensive iterative process is started leading to the generation of the data for the DFA diagrams. The strategy of selecting only the sensors with the highest discriminant power does not always lead to good group separation on the DFA diagrams, therefore, several attempts are made before deciding the discriminant power up to which peaks (sensors) are selected.

**Figure 1:**
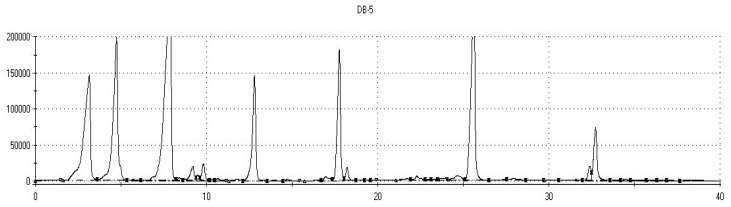

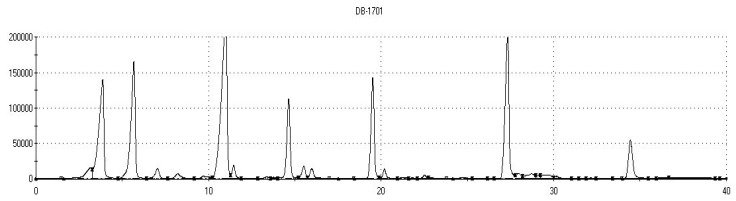
Example of chromatograms recorded by the Heracles e-Nose on its 2 columns (DB5 and DB1701 columns) for Feteasca regala control sample (G00_A00_T00).

An example of chromatograms obtained with the fast GC e-nose on both columns is provided in Figure 1, for the control sample. The others are very similar in appearance. The chromatograms for the same category of products are usually, at first sight, undistinguishable from one another, as the differences between samples differ only slightly, in the minor chromatographic peaks. This is also the case for our wine samples treated with various antioxidants, as the experimental samples are all prepared from the same grape must.

**Figure 2:**
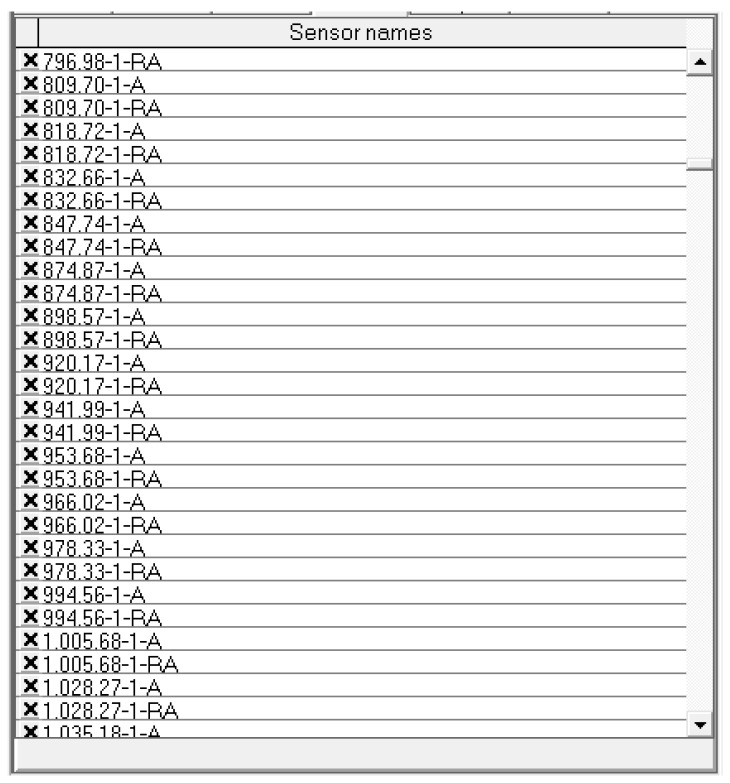
List of the sensors provided by electronic nose software after the process of integration and calibration.

These differences between chromatograms can only be assessed by a powerful statistical analysis, which the software of the e-nose, AlphaSoft is able to perform. By overlapping all wine sample chromatograms at once, AlphaSoft integrates them and creates a library of data. By also comparing the data with the chromatogram obtained in the same conditions for the standard alkane mixture (n-C6 to n-C16) calibration is achieved and Kovats indices are calculated. The results are provided by the software as a long list of codified sensors (Figure 2) together with their calculated values for the peak area A and peak relative area RA.

**Figure 3:**
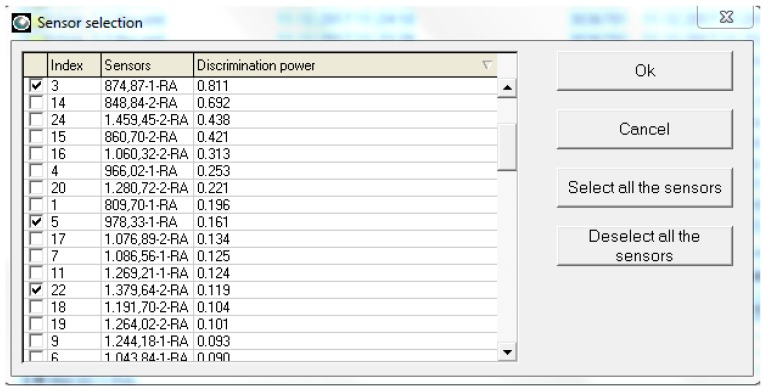
Sensor selection based on their discrimination power.

Because the list of sensors in Figure 2 is too extensive, a selection of the most relevant ones must be made, before the process of group sample discrimination by multivariate statistical analysis is started. The AlphaSoft determines and suggests the most discriminant chromatographic peaks for the samples grouped on treatment categories, but also calculates a “discrimination power” for each peak to facilitate the sensor selection (Figure 3). Based on our observations and trials, in order to obtain good group discrimination (high score validated classification of groups) the selection of sensors should include more peaks than those initially suggested by the software, the entire process of sensor selection being an iterative one.

Finally, many of the peaks selected as sensors can also be identified using their Kovats indices and available databases. In our study, most of the selected peaks were determined to correspond to substances found in the AroChemBase library and correlated with important sensory effects in food products and wine. These compounds were listed and used hereafter to differentiate the effect of several antioxidant treatments.

## 3. Results

The relevant volatile compounds of Feteasca regala are identified as described in the methodology with the use of the AroChemBase database and are presented in detail in Table 3. Sensory descriptors associated with the specific volatile organic compound are based on the information provided by AroChemBase and other open access databases, such as ChemSpider.

The substances included in Table 3 are all those possible to be identified by using the fast GC electronic nose’s compound database and they can be present in different amounts and combinations in the experimental wine samples analyzed. However, their quantification is not performed by the fast GC tool, which, working on the principle of the electronic nose, is only discriminating samples based on differences in their volatile organic compound combinations (wine fingerprints). This is one of the advantages of using this e-nose technology, that conclusions can be drawn in a faster and cheaper way, without having to invest in GC with mass spectroscopy to quantify the identified compounds. Thus, these substances/peaks were identified only to determine if they are more correlated than others to a certain antioxidant wine treatment. The correlation of a certain treatment with several wine volatile substances is easier to observe in the DFA bi-plots, which separate the groups of samples, but also show the compounds most likely to induce the separation.

Thus, discriminant factor analysis was applied to the values of relative area (RA) of certain chromatographic peaks (e-nose sensors in this case), selected by the procedure described in Material and Methods section.

The discrimination analyses for the groups of samples measured after one year of storage were not able to show statistical differences between the experimental variants with or without CO_2_ and with or without catechinic tannins additions respectively, as far as the volatile organic compound profile was concerned. For this reason, the samples were classified in larger groups of samples only based on the other treatments, as shown in Table 1: group I with GSH and AA, group II with GSH only, and group III with AA only.

**Figure 4:**
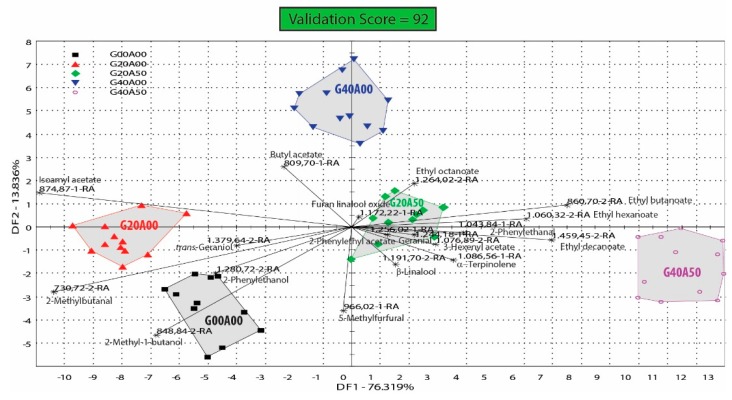
Discriminant factor analysis (DFA) bi-plot of groups of Feteasca regala wine samples treated with reduced glutathione alone (doses of 20 and 40 mg/L—G20A00, G40A00) or in combination with 50 mg/L ascorbic acid (G20A50, G40A50); G00A00 is the control not treated with glutathione or ascorbic acid.

Discriminant factor analyses were performed for groups of samples containing GSH, alone and in combination with doses of AA and the results are included in Figure 4.

As we can observe in Figure 4, the analyzed groups of samples (G00A00, G20A00, G40A00, G20A50, and G40A50) are well discriminated and separated on the DFA bi-plot, based on the differences in concentration of their volatile compounds, determined as relative area of the detected peaks on the chromatograms recorded on both columns of the e-nose. This DFA bi-plot shows that the first two dimensions explained 90.16% of the total variance observed over the samples, with 76.32% and 13.84% of the data variance explained by DF1 and DF2, respectively.

**Figure 5:**
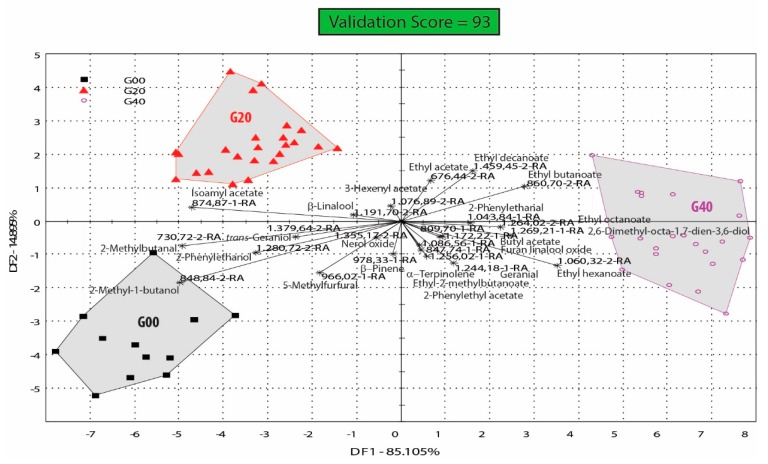
DFA bi-plot of groups of Feteasca regala wine samples treated with reduced glutathione (doses of 20 and 40 mg/L—G20, G40) with or without ascorbic acid; G00 is the control not treated with glutathione or ascorbic acid.

For a better understanding of reduced glutathione impact on volatile profile of the Feteasca regala wines, DFA analysis was also performed on the groups of samples containing 20 and 40 mg/L GSH (G20, G40) and the control wine group (G00). The G20 and G40 groups contained samples treated with glutathione, with or without addition of ascorbic acid. As we can observe in Figure 5, the three analyzed groups (G00, G20, and G40) are well discriminated and separated on the DFA bi-plot. In Figure 5, the first two dimensions explained 100% of the total variance, with 85.1% and 14.9% explained by DF1 and DF2 respectively.

**Figure 6:**
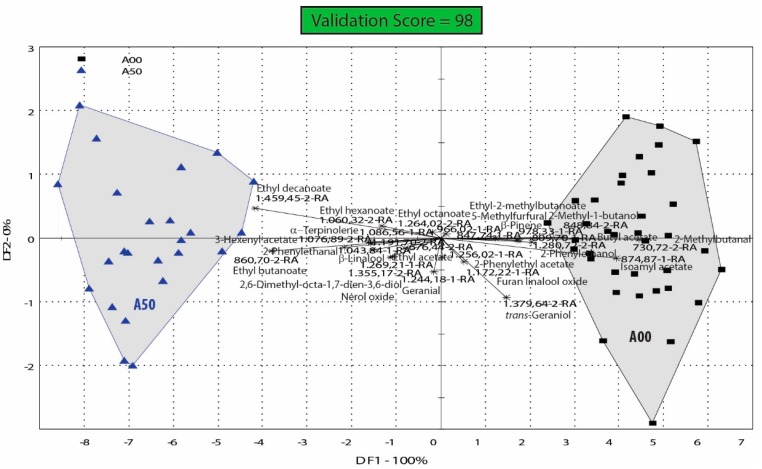
DFA bi-plot of groups of Feteasca regala wine samples treated with ascorbic acid of 50 mg/L (A50) with or without glutathione; A00 is the control not treated with ascorbic acid, with or without glutathione.

In order to understand the effect of ascorbic acid (AA) on the volatile organic profile of the resulted wines, samples have also been grouped and analyzed in accordance only with the presence or the absence of ascorbic acid treatment (Figure 6). As results, in Figure 6, we have only two groups (A00 and A50), which, logically, are well discriminated and separated on the DFA bi-plot, the total variance being explained entirely by the first dimension (DF1 = 100%). The use of this figure, however, is that it shows the main volatile compounds associated more with the treatment of the must with ascorbic acid.

## 4. Discussion

### 4.1. Glutathione and Ascorbic Acid Influence on the Aroma Profile of Feteasca Regala Wines

As it can be seen in Figure 4, control samples (without GSH or AA, but some with addition of tannin and/or carbon dioxide) included in group G00A00 were associated more with 2-phenylethanol and 2-methyl-1-butanol, 2-methylbutanal, 5-methylfurfural and to a lesser extent with *trans*-geraniol. The samples treated with 20 mg/L reduced glutathione, included in group G20A00, were relatively close to the control samples, but they contain more positive aroma compounds, such as isoamyl acetate, butyl acetate, *trans*-geraniol and to a lesser extent some more 2-methylbutanal and 2-phenylethanol. Wine samples treated with a higher dose of 40 mg/l reduced glutathione, included in group G40AA00 are associated with aroma compounds such as butyl acetate, furan linalool oxide, ethyl octanoate and, to a lesser extent, to isoamyl acetate. Wine samples treated with 20 mg/L reduced glutathione and 50 mg/L ascorbic acid (group G20A50) are linked with a better preservation of varietal aroma compounds such as β-linalool, α-terpinolene, geranial, and furan linalool oxide, along with fermentation aroma compounds such as 2-phenylethyl acetate, 2-phenylethanal, and 3-hexenyl acetate. The samples treated with 40 mg/L GSH and 50 mg/L AA (group G40A50) have also well preserved varietal aroma compounds, but they also contain considerable amounts of ethyl esters of fatty acids (ethyl butanoate, ethyl hexanoate, ethyl octanoate, and ethyl decanoate) formed during alcoholic fermentation, which have their particular contribution to fruity fresh aroma of these wines.

In general, control samples or group G00A00 are more associated with higher alcohols, aldehydes and furans, while the samples treated with only glutathione, groups G20A00 and G40A00, are more linked with acetate esters (butyl acetate, isoamyl acetate). Ascorbic acid addition in samples treated with GSH preserves better the terpenic compounds specific of this variety and also protects other aroma compounds from oxidation, many being still found in their aldehydic form.

These results highlight the main differences in the volatile profile associated with reduced glutathione treatments of the Feteasca regala musts and the major influence that ascorbic acid induces when added also along with the glutathione.

### 4.2. Glutathione Influence on the Aroma Profile of Feteasca Regala Wines

In Figure 5 samples were separated in groups only based on the presence of a certain dose of glutathione, irrespective of other antioxidant additions. In this case too, the control samples included in group G00 were associated mostly with the same volatile compounds as previously found in Figure 4, where the same group was used for comparison. This Figure 5, however, comparing groups containing a specific dose of glutathione, irrespective of the presence of treatments with ascorbic acid, allows for a better understanding of the GSH influence on the aroma profile of wines. Samples treated with 20 mg/L reduced glutathione irrespective of the ascorbic acid presence (group G20) are associated with more qualitative aroma compounds than control samples (group G00). The volatile organic compounds associated with the wines included in group G20 are isoamyl acetate, β-linalool and 3-hexenyl acetate. On the other hand, samples treated with 40 mg/L reduced glutathione are more complex, being associated with more volatile organic compounds such as ethyl esters of fatty acids (acetic, butyric, 2-metilbutanoic, hexanoic, octanoic, decanoic), acetate esters of higher alcohols (2-phenylethanol and butanol), aldehydes of higher alcohols (2-phenylethanal), monoterpenes and their related aldehydes (α-terpinolene, furan linalool oxide, and geranial).

### 4.3. Ascorbic Acid Influence on the Aroma Profile of Feteasca Regala Wines

As it can be observed in Figure 6, samples without ascorbic acid (A00 group) were associated more with higher alcohols (2-methyl-1-butanol and 2-phenylethanol), aldehydes (2-methylbutanal, 5-methylfurfural), certain esters (isoamyl acetate and butyl acetate, ethyl 2-methylbutanoate) and monoterpenes (*trans*-geraniol, furan linalool oxide and β-pinene). On the other hand, samples treated with 50 mg/L of ascorbic acid (A50 group) were associated more with ethyl esters of fatty acids (ethyl acetate, ethyl butanoate, ethyl hexanoate, ethyl octanoate, ethyl decanoate), monoterpenes and related aldehydes (β-linalool, α-terpinolene, nerol oxide, 2,6-dimethyl-octa-1,7-dien-3,6-diol and geranial) and other compounds such as 2-phenylethanal and 3-hexenyl acetate, confirming the observation from the DFA analysis included in Figure 4, that ascorbic acid preserves better the aroma compounds from oxidation.

## 5. Conclusions

Different treatments with antioxidants during vinification of Feteasca regala variety showed some distinctions regarding volatile organic compounds due to their effect on alcoholic fermentation and during wine aging in bottle. All of the identified volatile compounds are known as specific for the aroma of wines and their overall quality. Some of the identified compounds are specific for primary aroma, which is linked to the grape variety used, while others are formed during the alcoholic fermentation by the yeast intervention. For the wines of this study, the presence of terpenic compounds can clearly be linked to the Feteasca regala variety used. The other compounds, however, may be both from fermentation (biochemical reactions) or the result of wine evolution during storage in bottle (chemical reactions), all these interactions generating the final aroma of wine. The treatments with antioxidants on the must, before fermentation, have clear influences on the varietal aroma preservation in the produced wines, but can also have influences later on, on the evolution of wine during aging. These influences were found to vary in accordance to the type of antioxidants used and with their dosage.

The wines produced in the absence of antioxidant treatments showed a simple aromatic profile, with higher concentrations of some specific compounds generating basic wine aroma such as 2-methyl-1-butanol, 2-methylbutanal and some trans-geraniol hints specific for the grape variety. Other compounds such as 5-methyl furfural seem to be associated mostly with samples without ascorbic acid and without glutathione, this being a compound linked to oxidation.

The glutathione treatments induced more complexity in wines, the complexity increasing with the dose. The samples treated with 20 mg/L glutathione have aromatic profiles relatively close to those of the non-treated samples, showing however some improvement in the aromatic profile with more isoamyl acetate, butyl acetate and trans-geraniol than the control. Wine samples treated with 40 mg/L glutathione preserved a similar aromatic profile as the samples with 20 mg/L, but the floral notes induced by some more terpenic compounds intensified, among these specific compounds being the furan linalool oxide.

Ascorbic acid treatment is also beneficial for the final aroma profile of the wine, especially in combination with glutathione. A dose of 50 mg/L ascorbic acid leads to a better preservation of varietal aroma compounds such as β-linalool, α-terpinolene, geranial, and furan linalool oxide. With glutathione too, the aroma profile becomes more complex, with the addition of more notes from 2-phenylethyl acetate, 2-phenylethanal, 3-hexenyl acetate (with 20 mg/L glutathione) and more ethyl esters of medium chain fatty acids (with 40 mg/L glutathione).

## Figures and Tables

**Table 1:** Sample codes and antioxidant treatments.

Sample No.	Sample Code	Glutathione (mg/L)	Ascorbic Acid (mg/L)	Catechinic Tannin (mg/L)	Carbon Dioxide	Classification of Analyzed Groups		
	Additive Coding	G	A	T	CO_2_	Groupe	Groupe	Groupe
	Time of Addition	Winemaking		Bottling		I	II	III
1	G00_A00_T00	0	0	0	no	G00A00	G00	A00
2	G00_A00_T20	0	0	20	no			
3	G00_A00_T00_CO_2_	0	0	0	yes			
4	G00_A00_T20_CO_2_	0	0	20	yes			
5	G20_A00_T00	20	0	0	no	G20A00	G20	A00
6	G20_A00_T20	20	0	20	no			
7	G20_A00_T00_CO_2_	20	0	0	yes			
8	G20_A00_T20_CO_2_	20	0	20	yes			
9	G20_A50_T00	20	50	0	no	G20A50	G20	A50
10	G20_A50_T20	20	50	20	no			
11	G20_A50_T00_CO_2_	20	50	0	yes			
12	G20_A50_T20_CO_2_	20	50	20	yes			
13	G40_A00_T00	40	0	0	no	G40A00	G40	A00
14	G40_A00_T20	40	0	20	no			
15	G40_A00_T00_CO_2_	40	0	0	yes			
16	G40_A00_T20_CO_2_	40	0	20	yes			
17	G40_A50_T00	40	50	0	no	G40A50	G40	A50
18	G40_A50_T20	40	50	20	no			
19	G40_A50_T00_CO_2_	40	50	0	yes			
20	G40_A50_T20_CO_2_	40	50	20	yes			

**Table 2:** Instrument running conditions.

GC Run Conditions	Description
Analytical columns	**DB5 (non-polar):** 5% diphenyl, 95% dimethylpolysiloxane; **DB1701 (low/mid polarity):** 14% cyanopropylphenyl, 86% dimethylpolysiloxane.
Injection mode	2.5 mL HS syringe, extraction of volatiles from the head-space of vials
Injector temperature	250 °C
Temperature program	Initial column temperature 40 °C, increase rate of a 5 °C/s up to 200 °C
Carrier gas	Hydrogen in constant pressure mode, 16 psi
Oven temperature	10 min at 60 °C and 500 rpm
Acquisition time	46 s per sample; 5 min break between two samples
**Tenax trap conditions**	
Sampling temperature	40 °C
Desorption temperature	250 °C
Purge time	50 s
Bake-out time	50 s
**FID run conditions**	
FID temperature	220 °C
FID fuel pressure	35 psi

**Table 3:** Relevant volatile organic compounds identified in wine samples of Feteasca regala.

* Retention Time	GC Column	* Sample Kovats Indices	Database Kovats Indices	** Sensor Label	Identified Volatile Organic Compounds	*** Sensory Descriptors
**Aldehydes**						
7.12	DB1701	730.72	729	730.72-2	2-Methylbutanal	nutty, caramel, sweet
16.70	DB5	966.02	967	966.02-1	5-Methylfurfural	sweet, caramel, almond
19.84	DB5	1.043.84	1043	1.043.84-1	2-Phenylethanal	honey-like, apple, vegetable
**Higher alcohols**						
11.13	DB1701	848.84	852	848.84-2	2-Methyl-1-butanol	fruity, onion
28.14	DB1701	1.280.72	1282	1.280.72-2	2-Phenylethanol	roses, honey, sweet
**Ethyl esters of fatty acids**						
5.74	DB1701	676.44	673	676.44-2	Ethyl acetate	ethereal, anise, pineapple
11.88	DB5	847.74	850	847.74-1	Ethyl 2-methylbutanoate	apple, green, plum
11.59	DB1701	860.70	860	860.70-2	Ethyl butanoate	banana, ethereal, pineapple
19.68	DB1701	1.060.32	1061	1.060.32-2	Ethyl hexanoate	apple, banana, pineapple
27.53	DB1701	1.264.02	1260	1.264.02-2	Ethyl octanoate	pear, fruity, fresh
34.69	DB1701	1.459.45	−	1.459.45-2	Ethyl decanoate	grape, pear, oily
**Acetate esters**						
10.37	DB5	809.70	810	809.70-1	Butyl acetate	fruity, tropical, ethereal
12.98	DB5	874.87	874	874.87-1	Isoamyl acetate	banana, fruity, sweet
20.35	DB1701	1.076.89	1080	1.076.89-2	3-Hexenyl acetate	fresh, green, apple
28.01	DB5	1.256.02	1257	1.256.02-1	2-Phenylethyl acetate	floral, pollen
**Monoterpenes**						
17.25	DB5	978.33	978	978.33-1	β-Pinene	pine, turpentine, resin
21.54	DB5	1.086.56	1087	1.086.56-1	a-Terpinolene	herbal, pine, lemon, sweet
24.80	DB1701	1.191.70	1195	1.191.70-2	β -Linalool	citrus, floral, sweet
24.82	DB5	1.172.22	1166	1.172.22-1	Furan linalool oxide	earthy, floral, sweet
28.47	DB5	1.269.21	1272	1.269.21-1	2,6-Dimethyl-octa-1,7-dien-3,6-diol	sweet, floral, citrus
27.53	DB5	1.244.18	1243	1.244.18-1	Geranial	lemon, sweet
30.90	DB1701	1.355.17	1353	1.355.17-2	Nerol oxide	sweet, fruity, floral, rose
31.79	DB1701	1.379.64	1376	1.379.64-2	*trans*-Geraniol	sweet, apple, apricot, rose

* average values resulted from 3 recorded chromatograms (repetitions of the same sample); ** the sample label consists of the Kovats index and the column on which the chromatogram was recorded (1 = DB5; 2 = BD1701). *** The sensory descriptions for the identified compounds are taken from AroChemBase and other public databases.
